# Institutional Notes and News

**Published:** 1911-03-11

**Authors:** 


### Institutional Notes and News.

**The Happy Family at Bristo .**

Inasmuch as considerable satisfaction was expressed at the last annual Board meeting touching the improved state of the finances of the Bristol Royal Infirmary under the auspices of the president and treasurer, Sir George White, it may be mentioned that whereas statistics afforded locally in 1890 show that some twenty years ago the average annual expenditure was about £12,000, it had increased for the past year to £17,282, an increase perfectly justified by results. Sir George White's enthusiasm has been infectious, and, moreover, both at the Bristol Royal Infirmary and the Bristol General Hospital, the boast that has been made repeatedly is one for which there have been good grounds, and that boast is that the members of the management have had good reason for asserting themselves "a happy family."

**The Cancer Hospital (Free), Fulham Road, London, S.W.**

The sixtieth annual meeting of the Governors of this charity was held in the Board-room of the hospital on Wednesday, February 22, the Right Hon. the Earl of Northbrook, President, in the chair. From the report of the committee it appeared that during the year 1910 there were 665 new in-patients, whilst the average number of beds daily occupied was 85. In the Out-patient Department 1,789 new cases were treated, the total number of attendances for the year being 14,068. Reference was made to the great loss the charity had sustained by the death of Mr. Owen E. Hayter, who had been chairman since 1906, and also by the death of Mr. Charles R. Keyser and Mr. Cecil H. Leaf, two members of the senior honorary staff. The committee reported the erection of the Research Institute on a site adjoining the hospital grounds. This institute will shortly be fully equipped and ready to play a useful part in the great fight against cancer. It will be provided with separate laboratories and appliances for the careful and scientific investigation of the disease from all points, and the committee express the hope that adequate support will be forthcoming to help bear this additional burden. The Electrical and Radio-Therapeutic Department to the hospital was also claiming a large expenditure so as to bring the department up to date and allow those in charge to efficiently treat those cases placed under their care.

**Queen Charlotte s, Marylebone Road, N.W.**

The annual meeting of Governors and subscribers of Queen Charlotte's Lying-in Hospital was held on Tuesday, February 28, Mr. Frederick W. Hunt (Vice-President) in the chair. During the year 1,775 women had been admitted to the wards of the hospital, and 2,449 others had been attended and nursed in their own homes. The report referred to the very low rate of mortality, namely, 3.3 per thousand in the case of the in-patients and 0.4 per thousand in the case of the out-patients. A grant of £1,500 had been received from King Edward's Hospital Fund, £500 of which was towards improvements recently carried out, including the installation of electric light and electric sterilisers. The Chairman stated that the income had shown some improvement over recent years, and the committee had been able to clear off the deficit of £3,287 incurred during the years 1907, 1908, and 1909. There was, however, still a debt of £2,130 on the Nurses' Home, which the committee were anxious to pay off. The work done by the hospital in training medical students, midwives and monthly nurses steadily increased, and the applications received at the hospital for maternity nurses for private cases showed that the establishment of the private nursing staff was greatly appreciated.

**Grantham Hospital.**

Mr. T. S. Pearson Gregory presided at the thirty-sixth annual meeting of the Grantham Hospital, when it was reported that 235 patients had been received during the year, an increase of 11; the operations performed were 140, and the anæsthetics administered 155. The average stay per patient was 26.69 days. The income was £1,660, and the expenditure £1,411 1s. 11d. A carbon dioxide snow apparatus had been given to the Institution by the Hon. Mrs. Anderson Pelham, Mr. J. V. Hemery and Dr. G. A. C. Shipman. The Committee of Management was reappointed as follows: Sir Charles G. E. Welby, Bart., C.B., the Bishop of Grantham, Mr. E. Grinling, Mr. Tryner Lynn, Mr. Wm. Laud, Mr. J. W. Cole, Mr. W. Bradshaw; and the Vicar of Grantham and Mr. J. Bowman were elected to fill vacancies. The Institution has benefited by a legacy of £50 from the late Miss M. A. Roberts.

**Beckett Hospital, Barnsley.**

The extensions and improvements at this hospital are progressing very satisfactorily. The additions and improvements now completed include a new electric laundry, which has cost £1,300; new casual, *x*-ray room, and out patients' department, £1,200; improvements to operating theatre and ophthalmic department, £400; and additions to heating apparatus, painting, and decorating, £650. In addition to above, contracts have been accepted, and work is in progress, for erecting a new wing, a new operating theatre, new kitchen, store-rooms, etc., which will entail a further expenditure of over £10,000. This amount, together with items named above, means an expenditure of over £14,000, and when the work is completed Barnsley will have one of the most up-to-date voluntary hospitals (102 beds) in the district, and will be a fitting memorial of the Coronation year. We are pleased to note that the appeal made to its friends has been very generously responded to, £9,000 having already been subscribed towards the £14,000. A very splendid act of devotion to voluntary hospitals has been recently agreed upon by the working-men members of the Barnsley Co-operative Society. At their annual meeting held last week in the Public Hall they unanimously agreed to pay the entire cost of providing the proposed new operating theatre, along with the anæsthetic and recovery rooms attached. This will relieve the committee of over £1,000 liability. In addition to this, and in order to perpetuate the celebration of the jubilee of their Society, they further propose to endow a bed in commemoration of their success during the past 50 years. A decision like this speaks well for the good feeling shown to voluntary hospitals by the working classes, and might be copied with advantage in other centres of industry. The Saturday and Sunday Committee is also giving financial help to the improvements, and during the past two years subscribed £850 towards the scheme. This committee, which is entirely composed of working men, contributes each year half of the income for the upkeep of the hospital. Another feature at the institution is the interest taken in the children's ward by the elementary school children. They have a Children's League, and contribute at least 1d. each year as a donation for its maintenance. They raise about £30. The amount is not much, but the educational influence bears fruit in after years, in inculcating a disposition to help the sick.

